# ITGB4 deficiency in bronchial epithelial cells directs airway inflammation and bipolar disorder-related behavior

**DOI:** 10.1186/s12974-018-1283-5

**Published:** 2018-08-31

**Authors:** Li Han, Leyuan Wang, Sha Tang, Lin Yuan, Shuangyan Wu, Xizi Du, Yang Xiang, Xiangping Qu, Huijun Liu, Huaiqing Luo, Xiaoqun Qin, Chi Liu

**Affiliations:** Department of Physiology, School of Basic Medical Science, Xiangya School of Medicine, Central South University, Changsha, Hunan 410007 People’s Republic of China

**Keywords:** ITGB4, Bipolar disorder (BD), Mania, Depression, Microglia, Inflammation

## Abstract

**Background:**

Chronic persistent airway inflammation has been associated with the comorbidity of asthma and bipolar disorder (BD). However, the direct relevance between airway inflammation and BD-like psychiatric comorbidity is almost unknown. Integrin β4 (ITGB4) is downregulated on the airway epithelial of asthma patients, which might play a critical role in the parthenogenesis of airway inflammation. So this study aimed to examine the role of ITGB4 deficiency in mediating airway inflammation and further leading to the BD-like behaviors.

**Methods:**

ITGB4^−/−^ mice were generated by mating ITGB4^fl/fl^ mice with CCSP–rtTA^tg/−^/TetO-Cretg/tg mice. Mania-like behavior tests were performed, including hyperlocomotion, d-amphetamine-induced hyperactivity, open-field test, and elevated plus-maze test. Depressive-like behavior tests were carried out, including sucrose preference, forced swimming, and learned helplessness. Inflammatory cells (Th17, Th1, Th2) in the lung were examined by flow cytometry. Futhermore, inflammatory cytokines (IL-4, IL-13) in bronchoalveolar lavage fluid and sera were detected by ELISA. Protein expression of the IL-4Rα on choroid plexus, microglial marker (IBA1), and synapse-associated proteins (synaptophysin, SYP) in the hippocampus and prefrontal cortex were examined by western blotting. Additionally, proinflammatory cytokines (IL-1β, IL-6, and TNF-α) in the hippocampus and prefrontal cortex were detected by immunohistochemistry. Inflammatory disorder in the lung, hippocampus, and prefrontal cortex was tested by hematoxylin and eosin (H&E) staining. And cell apoptosis in the hippocampus and prefrontal cortex was measured by TUNEL test.

**Results:**

ITGB4^−/−^ mice exhibited mania-like behavior, including hyperlocomotion, d-amphetamine-induced hyperactivity, and reduced anxiety-like behavior. While under stressful conditions, ITGB4^−/−^ mice manifested depressive-like behavior, including anhedonia, behavioral despair, and enhanced learned helplessness. At the same time, ITGB4^−/−^ mice mainly exerted Th2-type inflammation in periphery, like the number and major cytokines IL-4 and IL-13 of Th2-type inflammation. ITGB4^−/−^ mice also showed a significant increase of microglia and pro-inflammatory cytokines such as IL-1β, IL-6, and TNF-α in the hippocampus and prefrontal cortex. Additionally, neuron damage, increased neuron apoptosis, and the decrease of SYP were found in ITGB4^−/−^ mice.

**Conclusions:**

These findings confirmed that airway inflammatory induced by ITGB4 deficiency is the important incentive for the BD-like behavior during asthma pathogenesis. The ITGB4-deficient mice provide a validated animal model for us to study the possible mechanism of BD-like psychiatric comorbidity of asthma patients.

## Background

Asthma is the most common chronic airway disease that has implied a much greater prevalence of mental disorders such as bipolar disorder (BD) [1, 2]. The association of asthma and psychiatric comorbidity indicated symptom severity [3, 4], poorer asthma control [5], and higher comorbidities and increased use of health services [6], leading to heavy socioeconomic burden [6, 7]. Thus, this clinical phenomenology has caused greater attention and concern from public health communities worldwide. However, to the best of our knowledge, the mechanism about the association between asthma and BD is not unequivocal.

Airway inflammation is the most important pathological feature of asthma [8], featuring the increased migration and activation of Th2 lymphocytes, mast cells, eosinophils, and macrophages [9, 10]. Inflammatory cytokines like interleukin-4 (IL-4), IL-5, and IL-13 are also increased and altered along with asthma exacerbation [11]. Notably, accumulating evidences have proved that dysfunction of inflammation occur in mental disorder patients [12]. IL-4, IL-6, and IL-12 may change in bipolar disorder (BD) patients under different mood episodes (mania or depression episode) [13, 14]. More and more related studies indicate that the immune system responses chronically activated by macrophages and T lymphocytes may result in mood dysregulation as the peripheral inflammation transmits information to the brain [15]. Consistent with this notion, microglia, the resident immune cells in the brain, might function as an important interface to transmit such information [16]. Moreover, peripheral immune system and inflammatory processes have demonstrated alteration in many patients with bipolar disorder [17]. Cytokines, connecting peripheral immune to central nervous systems [15], have shown altered levels in patients with bipolar disorder as compared with individuals without disorder [17]. However, few reports have examined the direct relevance between airway inflammation and its psychiatric comorbidity BD in asthma.

Our previous work found that ITGB4, a structural adhesion molecule, is downregulated in airway epithelial cells of asthma patients with four variation sites in 5′ flanking region [18, 19]. ITGB4, a heterodimeric transmembrane receptor, is located at the basal surface of airway epithelial cells in hemidesmosomal structures that function as structural link between epidermal cells and the underlying basement membrane [20, 21]. ITGB4 also regulates pathological airway conditions of inflammation responses through integrin-associated signaling and recruitment of adaptor molecules [22, 23]. In addition, ITGB4 leads to activation of the Rho GTPases [24] as well as MAPK [25], PI3-K [26], and NF-κB signaling [27] pathways which were highly relevant to the propagation of inflammation and injury. Meanwhile, the increased permeability of the bronchial epithelium to HDM has been associated with enhanced NF-κB activity and increased pro-inflammatory cytokine expression, which implies that the disruptions of airway epithelial barrier may have immunomodulatory consequences [28, 29].

Therefore, in the present manuscript, we utilized ITGB4 conditional knockout mice to investigate the direct induction of airway inflammation with BD and unravel the mechanisms that peripheral inflammation information is transmitted to the brain to affect the neuronal network and trigger BD-like behaviors.

## Methods

### Animals

The CCSP–rtTA^tg/−^/TetO-Cre tg/−/ITGB4 ^fl/fl^ triple transgenic mice [30] were generated by mating ITGB4^fl/fl^ mice [31] with CCSP–rtTA^tg/−^/TetO-Cre^tg/tg^ mice on a C57BL/6 background [32]. To produce ITGB4^−/−^ mice with ITGB4 conditionally knocked out in their airway epithelial cells, doxycycline (Dox; 1% in drinking water) was ingested from E7.5 to the end of experiment. ITGB4^fl/fl^ male littermates lacking either CCSP–rtTA, TetO-Cre, or both transgenes were used as control mice which were given identical dosage of doxycycline. Male mice with sexual maturity were used for the researches. The 5-min and 30-min open-field tests, as well as amphetamine challenge tests, were performed on the same cohort of mice. Another cohort of mice was tested in elevated plus-maze test, forced swim test, and learned helplessness test in turn on different days. Sucrose preference test were performed immediately after 30-min restraint stress using the third cohort of mice.

**Fig. 1:**
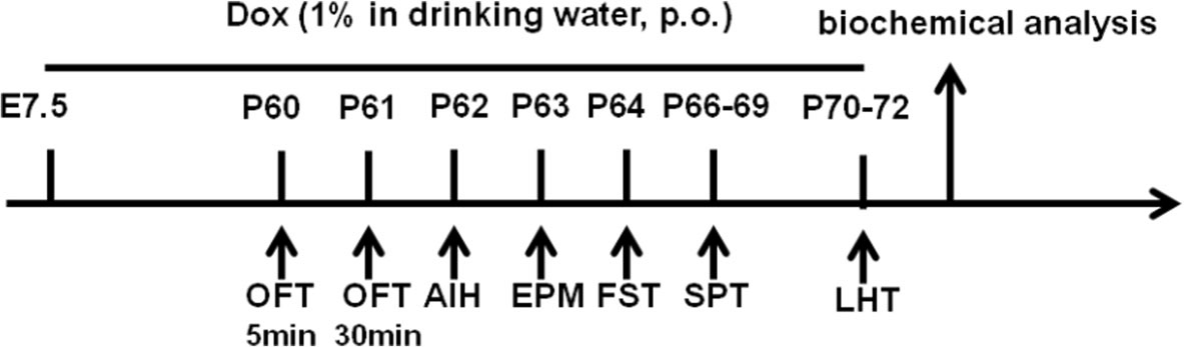
Schematic timeline of the experimental designs. Mice were given doxycycline with oral (Dox; 1% in drinking water) per day from E7.5 to the end of experiment. Open-field test (OFT), amphetamine-induced hyperactivity (AIH), elevated plus-maze (EPM), forced swimming test (FST), sucrose preference test (SPT), and learned helplessness test (LHT) were conducted on days 60, 61, 62, 63, 64, 66–69, and 70–72, respectively

The mice were maintained under a temperature and humidity controlled housing conditions with a 12:12 h light-dark cycle and free access to food and water. The mice were treated daily for 1 week to habituate them to the experimenter before behavioral testing, the schedule of which is showed in Fig. 1. All experimental protocols were carried out according to the National Institutes of Health Guide for the Care and Use of Laboratory Animals approved by the Central South University at XiangYa Animal Care and Use Committee.

### Behavioral tests

#### Forced swimming test (FST)

The forced swimming test for mice described by Porsolt was used to assess depression-like behavior [33]. In this test, mice were dropped individually into an inescapable Plexiglas cylinder (RWD Life Science Co., Ltd., Shenzhen, Guangdong, China), 30 cm tall, 11 cm diameter filled with 10 cm water (25 ± 1 °C). The sessions lasted for 6 min and behavior was recorded by a video camera in a dimly lit room illuminated with an indirect 15 Lux white light box, and then mice were removed from water, gently dried and placed into home cage. Water was changed between subjects. The immobility time during the last 4 min of the test session was measured to evaluate depressive-like behaviors by an expert observer.

#### Restraint stress

Mice suffered restraint stress according to established protocols. Stressed mice were placed in a ventilated 50-ml plastic Falcon tube with eight small (0.5 cm) air holes for 30 min. Control mice still remained in their home cage.

#### Sucrose preference test (SPT)

The SPT procedure was carried out on four consecutive days. During the first 2 days, two bottles were introduced to each cage, one containing 200 ml of 1% sucrose and the other containing 200 ml of tap water. The positions of sucrose and water bottles were switched every 12 h to eliminate side preference. Then, the two bottles were removed from the cages in the third day to ensure motivation to drink water. Immediately following the deprivation period, mice were subjected to either 30 min restraint or brief experimenter handling, returned to their home cage, and allowed to drink sucrose or water randomly. The volume of water and sucrose consumed during the 1 h was noted, and sucrose preference was measured as sucrose consumed/total liquid consumed.

#### Learned helplessness test (LHT)

The LH procedure involves two phases: shock pretraining and avoidance-escape testing, as described in Kole Roybal’s paper [34]. Briefly, each group mice were assigned to no-foot-shock (NFS) and foot-shock (FS) groups. During the shock pretraining, FS mice were placed in a chamber in which they received unsignalled inescapable foot (5 s duration at 0.3 mA) with a mean interval of 30 s (twice a minute) for 1 h and then returned to their home cage 30 s after the final shock, while NFS mice were allowed to explore the chamber for 1 h. The same procedure was conducted on day 2. During the avoidance-escape testing (on day 3), the automatic door opened concurrent with presentation of each foot shock (0.3 mA, 30 trials with 30–60 s inter-trial interval), allowing the mice to escape. Mice escaped to the non-shocked compartment were called an escape response, or if they failed to escape within 20 s, this was recorded as an escape failure, an indicator of depressive behavior. The numbers of escape failures and intertrial crossings were recorded during the test session.

#### Open-field test (OFT)

The OFT was performed in the apparatus consisted of a gray square 40 cm × 40 cm × 40 cm. The central area was defined as a 15 × 15 cm square, which had been marked on the floor. The mice were singly placed into the center of the floor, and after 30 s of accommodation, the total distance traveled and the time spent in the central section of the apparatus were recorded for 5 min and 30 min on two different days and analyzed by the Panlab Samrt v3.0 behavioral video tracking software (Panlab, Cambridge, USA). After each test, the open field was cleaned with 70% alcohol solution.

#### Amphetamine-induced hyperactivity (AIH)

The response to psychostimulants was tested for 30 min immediately after a single intraperitoneally (i.p.) injection of 2 mg/kg d-amphetamine sulfate (D-AMPH; Sigma-Aldrich) dissolved in saline at a volume of 4 ml/kg. The total distance traveled after D-AMPH challenge was calculated for assessing activity.

#### Elevated plus-maze test (EPM)

The EPM comprised of two enclosed (6 × 30 × 15 cm) and two open (6 × 30 cm) arms that stretch from a common central platform (6 × 6 cm) elevated 50 cm off the ground. Each mouse spent 5 min in the experimental apparatus. The test started by gently placing a mouse at the center of the maze facing an open arm. An entry was recorded when more than half of the mouse’s trunk into the arm. The time spent in the open arms and the number of entries to each arm were calculated. These behavioral data were automatically collected by a video-tracking system (Panlab Samrt v3.0, purchased from RWD Life Science Co., Ltd.).

### Isolation of sera

Mouse blood were withdrawn from sterile retro-orbital artery into a free-anticoagulant vacuum tube and centrifuged at 2000 rpm for 10 min. Serum was collected from the top layer in the tube and stored at − 80 °C for further experiments.

### Bronchoalveolar lavage fluid (BALF)

Mice were sacrificed by intraorbital arterial bleeding. As soon as ligating the left main bronchus, lavage the right lung using 0.4 ml saline, do this for three times. The supernatants of bronchoalveolar lavage fluid (BALF) were collected through 10 min of centrifugation at 4 °C and 1500 rpm and stored at − 80 °C for assay of BALF cytokines.

### Flow cytometry

Lung cell suspensions were obtained as previously described [35]. Briefly, lung cell suspensions were prepared by enzymatically digesting the lung tissue using 1.5 mg/ml of collagenase I in serum-free medium. Then, single-cell suspensions were stained with FITC-labeled anti-mouse CD4. After permeabilization, intracellular staining was performed with the addition of PE-labeled anti-mouse IL-17A (eBioscience, 12-7177), anti-mouse IL-4 (eBioscience 12-7311), and anti-mouse IFN-γ (eBioscience, 12-7311). Cells were counted by flow cytometry (FACS Calibur, BD Biosciences) and analyzed by CellQuest software, with CD4−IL-17A representing Th17 cells, CD4−IL-4 representing Th2 cells, and CD4−IFNγ representing Th1 cells.

### Western blotting

Western blot analysis was performed according to previously published procedures [36]. Tissue samples were harvested with RIPA lysis buffer containing 1% proteinase inhibitor cocktail (Sigma-Aldrich) according to protocols, and then centrifuged to collect supernatants. SDS Loading Buffer was added to supernatants, and then boiled before separated by 10% SDS-PAGE. After electrophoretically transferring protein to polyvinylidene difluoride membranes (Millipore), the membranes were incubated with primary antibodies, ITGB4 Abcam ab182120, 1:1000, synaptophysin (SYP) Millipore MAB5258-I, 1:1000, TNFRα Santa Cruz sc-8436, 1: 1000, IBA1 Santa Cruz sc-32,725, 1: 1000, and IL-4Rα Santa Cruz sc-28361, 1: 1000, and subsequently reacted with horseradish peroxidase-conjugated secondary antibody prior to visualizing through the use of ECL reagents (Pierce).

### Histology, H&E, immunofluorescence and immunochemistry

Brain and largest lobe of the left lung were inflated, fixed in 4% paraformaldehyde, and processed for paraffin embedding. Five-micrometer sections were stained with hematoxylin and eosin (H&E) as described previously [37]. Immunofluorescent staining was performed on mouse lung paraffin sections with the following antibodies: ITGB4 Abcam ab182120 1:200 and CCSP Santa Cruz sc-365992 1:200. IHC analyses were performed on mouse brain paraffin sections using IL-6 Santa Cruz sc-57315 1:200, TNFα Abcam ab6671 1:200, and IL-1β Santa Cruz sc-12742 1:200. Zeiss Axio Scope.A1 or Zeiss Discovery.V8 Stereo microscope (Carl Zeiss MicroImaging GmbH, Göttingen, Germany) was used and integrated with an Axio-Cam ICc3 camera (Spectra Service, Ontario, NY). Images were obtained by AxioVision Rel. 4.7 software from Zeiss.

### Enzyme-linked immunosorbent assay (ELISA)

The mouse bronchoalveolar lavage fluid (BALF) and sera were collected to measure the levels of the cytokines IL-4 (BioLegend) and IL-13 (BioLegend) using ELISA kits following the manufacturer’s guidelines.

### Apoptosis detection

According to TUNEL test kit instructions (Promega), the paraffin slice was hydrated through graded alcohols, 3% oxygen hydrogen was used to block endogenous horseradish peroxidase. After digestion by proteinase K for 15 min at room temperature, tissue slices were incubated by the mixed buffer of Biotinylated Nucleotide Mix and rTdT reaction mix at 37 °C for 60 min inside a humidified chamber and then incubated with HRP for 30 min at room temperature, and finally dyed by DAB. The dyeing sections were examined under light microscopy (Nikon).

### Statistical analysis

All data were performed with SPSS v19.0 and expressed as mean ± SEM. The *p* value < 0.05 was regarded as statistically significant. Differences between two groups were analyzed using Student’s *t* test, whereas significant differences among multiple groups were confirmed by two-way ANOVA followed by Tukey’s post hoc test.

## Results

### Silencing efficiency was detected in ITGB4^−/−^ mice

**Fig. 2:**
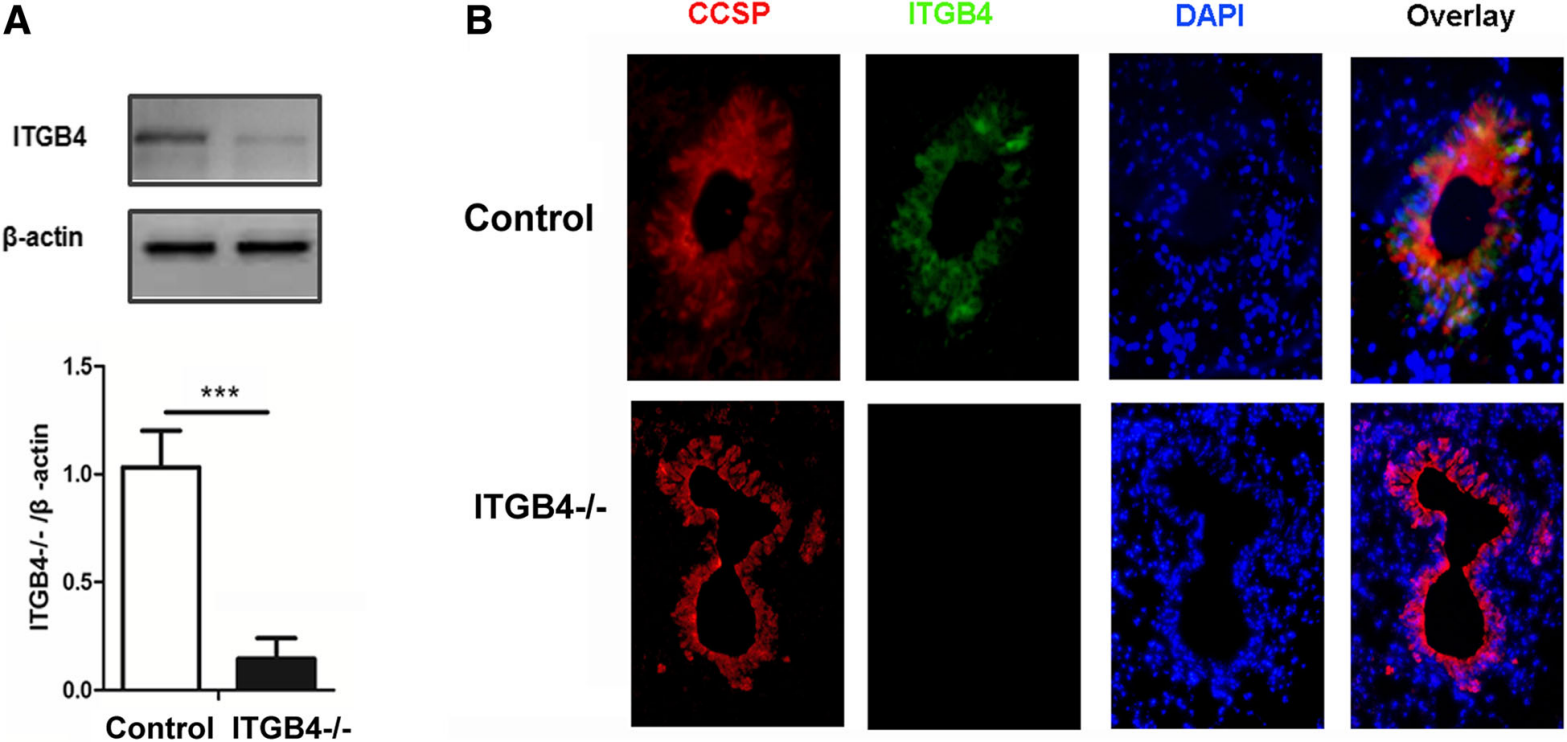
Silencing efficiency of ITGB4 was detected. **a** ITGB4 protein expression and quantification were determined by western blot in lung tissues. **b** ITGB4 expression was detected by immunofluorescence. Co-localization of CCSP (red) and ITGB4 (green) were performed in lung sections. DAPI was used to stain cell nuclei (blue). All images were obtained × 400 magnification. The data are expressed as the mean ± SEM of four mice in each group. ***p < 0.001

We confirmed that the model of ITGB4-deficient mice was established by the analysis of the ITGB4 expression changes in control mice and ITGB4^−/−^ mice. A conspicuous block in ITGB4 protein was detected from primary CCSP^+^ airway epithelial cells in ITGB4^−/−^ mice (Fig. 2a, *p* < 0.001). In addition, triple immunofluorescence staining was used to detect ITGB4 expression in airway epithelial cells. In control mice, ITGB4 was detected in near-linear basilar stained airway cells throughout the conducting airways. In ITGB4^−/−^ mice, ITGB4 expression was blocked significantly in the conducting bronchi and proximal bronchioles (Fig. 2b).

### Locomotor hyperactivity and increased sensitivity to amphetamine in ITGB4^−/−^ mice

**Fig. 3:**
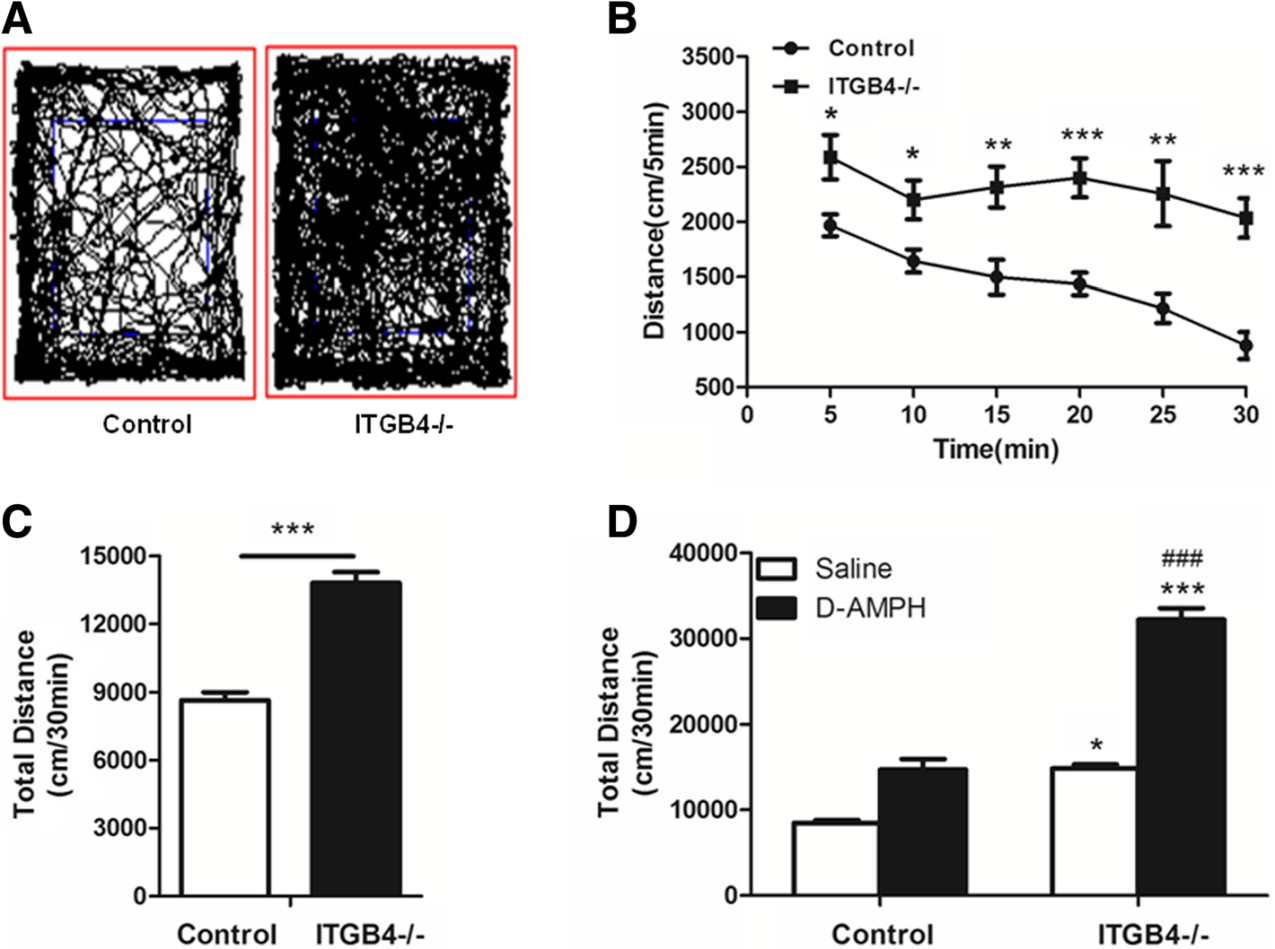
ITGB4^−/−^ mice show hyperlocomotion and enhanced sensitivity to D-AMPH in the open field. **a** Representative tracking paths for ITGB4^−/−^ and control mice during the 30-min open-field test (*n* = 9). **b** ITGB4^−/−^ mice showed overall hyperactivity during 30 min (5 min per point) test in the open-field (*n* = 9). **c** ITGB4^−/−^ mice traveled further in the 30-min open-field (*n* = 9). **d** Bar graph presented the total distance traveled during the 30-min monitoring period after D-AMPH injection (*n* = 9) .*p < 0.05, ***p < 0.001 vs saline in control group; ###*p* < 0.001 vs D-AMPH in control group. All data are presented as mean ± SEM. *p < 0.05, **p < 0.01, ***p < 0.001

**Fig. 4:**
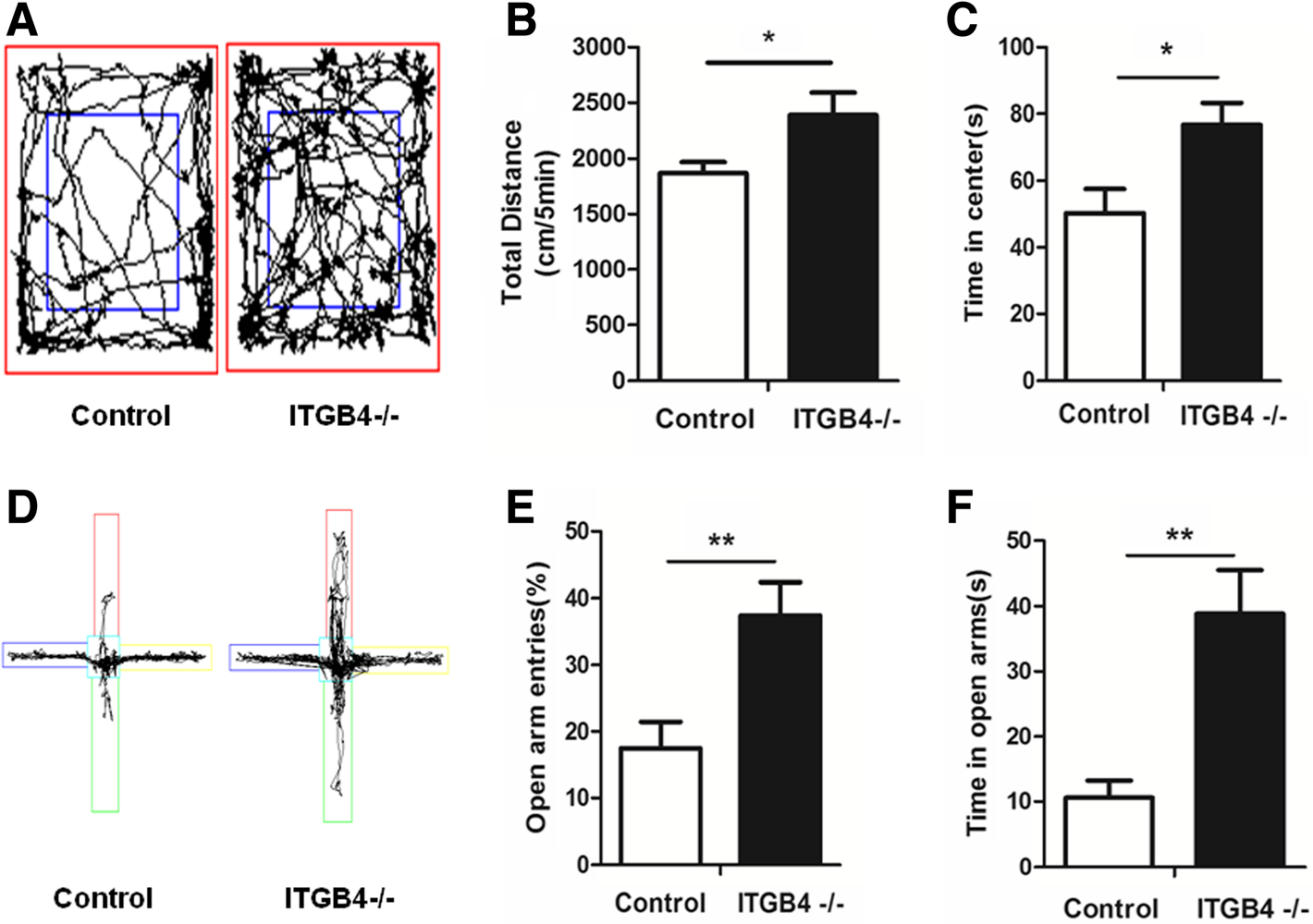
ITGB4^−/−^ mice show mania-like behavior. **a** Representative tracking data for ITGB4^−/−^ (*n* = 10) and control (*n* = 9) mice during the 5-min open-field test. **b**, **c** ITGB4^−/−^ mice traveled further in the 5-min open-field test and spent more time in the central area. **d** Representative tracking paths for ITGB4^−/−^ and WT mice during the elevated plus maze test (EPM). **e**, **f** ITGB4^−/−^ mice (*n* = 9) entered the open arms of the elevated plus maze more frequently and explored the open arm for a longer duration than control mice (*n* = 8). All data are presented as mean ± SEM. *p < 0.05, **p < 0.01

To assess exploratory behavior and locomotor activity, the same group of mice was subjected to 5-min (Fig. 4b) and 30-min (Fig. 3c) open-field tests on two different days. ITGB4^*−/−*^ mice were hyperambulate in both a novel environment (5-min open-field) (Fig. 4b, *p* < 0.05) and a familiar environment (30-min open-field) (Fig. 3a, c, *p* < 0.001), which eliminated novel environmental stimuli to lead to hyperactivity. The ITGB4^−/−^ mice exhibited hyperambulation over a period of 30 min, while control mice showed a time-dependent reduction in locomotion (Fig. 3b, genotype × time, *F* (1, 16) = 24.036, *p* < 0.05), suggesting a genotype difference in habituation.

Hyperactivity has been reported in patients and animal models of both attention-deficit hyperactivity disorder (ADHD) and mania [38–40], while d-amphetamine(D-AMPH) exacerbates hyperactivity in bipolar disorder, but decreases locomotor activity in ADHD. So, we confirm that the hyperactivity in ITGB4^−/−^ mice is related to mania or ADHD by testing the response to D-AMPH. Mice were treated with an acute injection of D-AMPH (2 mg/kg, i.p.), and locomotor activity was evaluated in an open field for 30 min. As expected, the D-AMPH injection evoked a greater degree of hyperactivity in ITGB4^−/−^ mice than control mice (Fig. 3d, genotype × treatment interaction, *F* (1,32) = 58.473, *p* < 0.001).

### Increased anxiolytic-like behavior in ITGB4^−/−^ mice

The core symptoms of mania are low level of anxiety, greater risk-taking and greater impulsivity. To assess the level of anxiety-like behavior, we used the 5-min open-field test (Fig. 4a–c) and the elevated plus maze test (Fig. 4d– f). In the 5-min open-field test, ITGB4^−/−^ mice showed significantly increased in the total traveling distance (Fig. 4a, b, *p* < 0.05) and spent more time in central area where the anxiety of animals could be easily provoked (Fig. 4c, *p* < 0.05).

In the elevated plus maze, ITGB4^−/−^ mice showed higher percentage of open arm entry (Fig. 4d, e, *p* < 0.01) and more time spent on (Fig. 4f, *p* < 0.01) the aversive open arms in comparison to control mice.

### Stress-induced depressive-like behavior in ITGB4^−/−^ mice

**Fig. 5:**
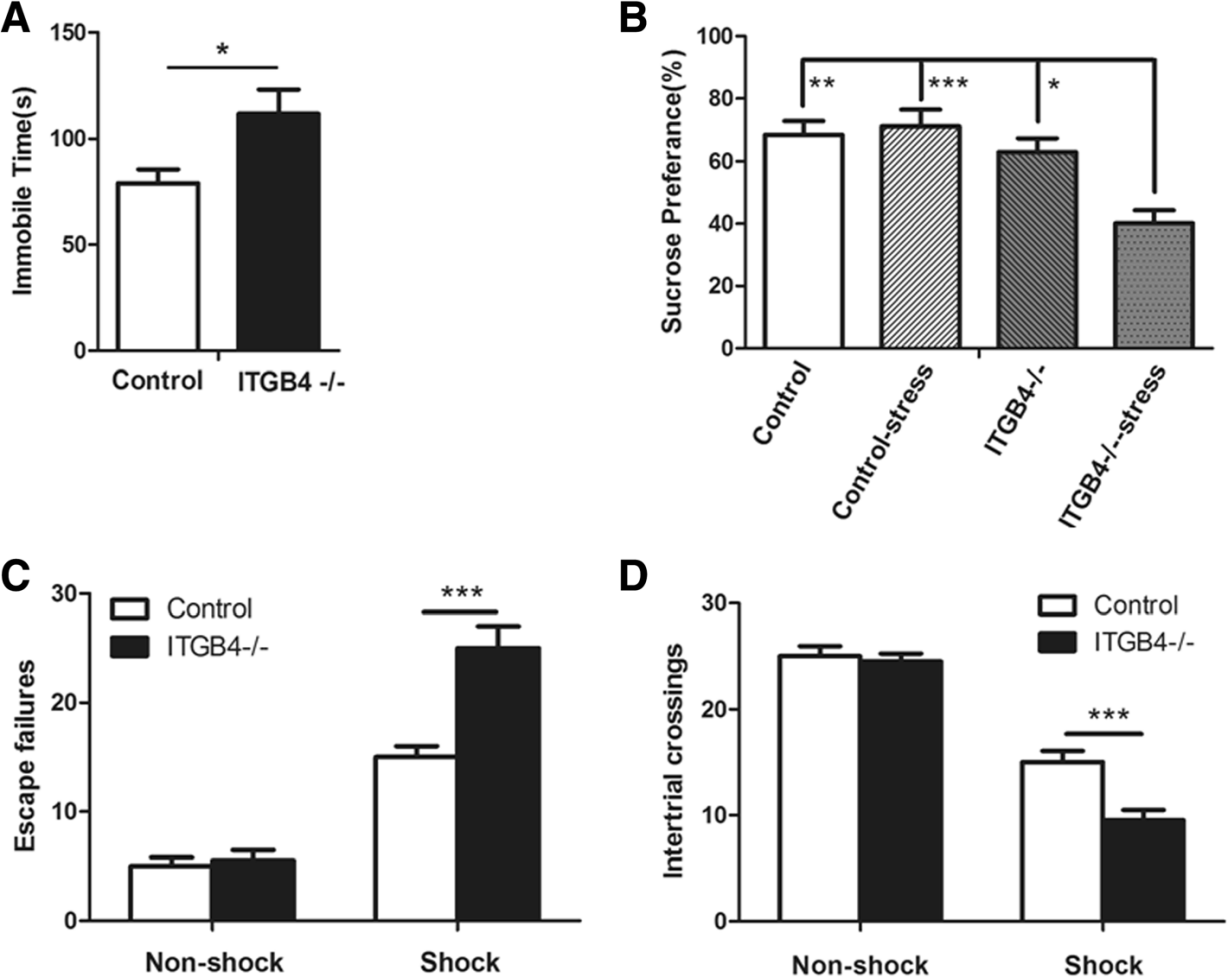
Stress-induced depression-like behavior in ITGB4^−/−^ mice. **a** ITGB4^−/−^ mice spent more time immobile in the forced swim test (*n* = 8). **b** Restraint stress for 30 min caused a drop of sucrose preference in ITGB4^−/−^ mice (*n* = 8). **c**, **d** ITGB4^−/−^ mice had more escape failures and a less crossing numbers in the learned helplessness test (*n* = 8). All data are presented as mean ± SEM. *p < 0.05, **p < 0.01, ***p < 0.001

Depressive-like behavior was quantified by forced swim test (FST). In this study, ITGB4^−/−^ mice showed higher levels of immobility time than control littermates (Fig. 5a, *p* < 0.05), suggesting an increase in despair behavior.

During the sucrose preference test, no significant difference in sucrose consumption was observed between control mice and ITGB4^−/−^ mice (Fig. 5b). However, ITGB4^−/−^ mice given restraint stress presented a significant decrease in sucrose consumption (Fig. 5b, genotype × stress interaction, *F* (1,28) = 9.418, *p* < 0.01).

The learned helplessness paradigm is a sub-chronic stress test of depressive-like behavior. The NFS mice presented few escape failures and more crossing numbers compared with the FS mice. There was no difference in escape failures and numbers of chamber crossings between control and ITGB4^−/−^ mice in the NFS group (Fig. 5c, d). However, in the FS group, ITGB4^−/−^ mice had a higher escape failures (Fig. 5c, *p* < 0.001) and a lower crossing numbers (Fig. 5d, *p* < 0.001) than control mice.

### Peripheral inflammation increase in ITGB4^−/−^ mice

**Fig. 6:**
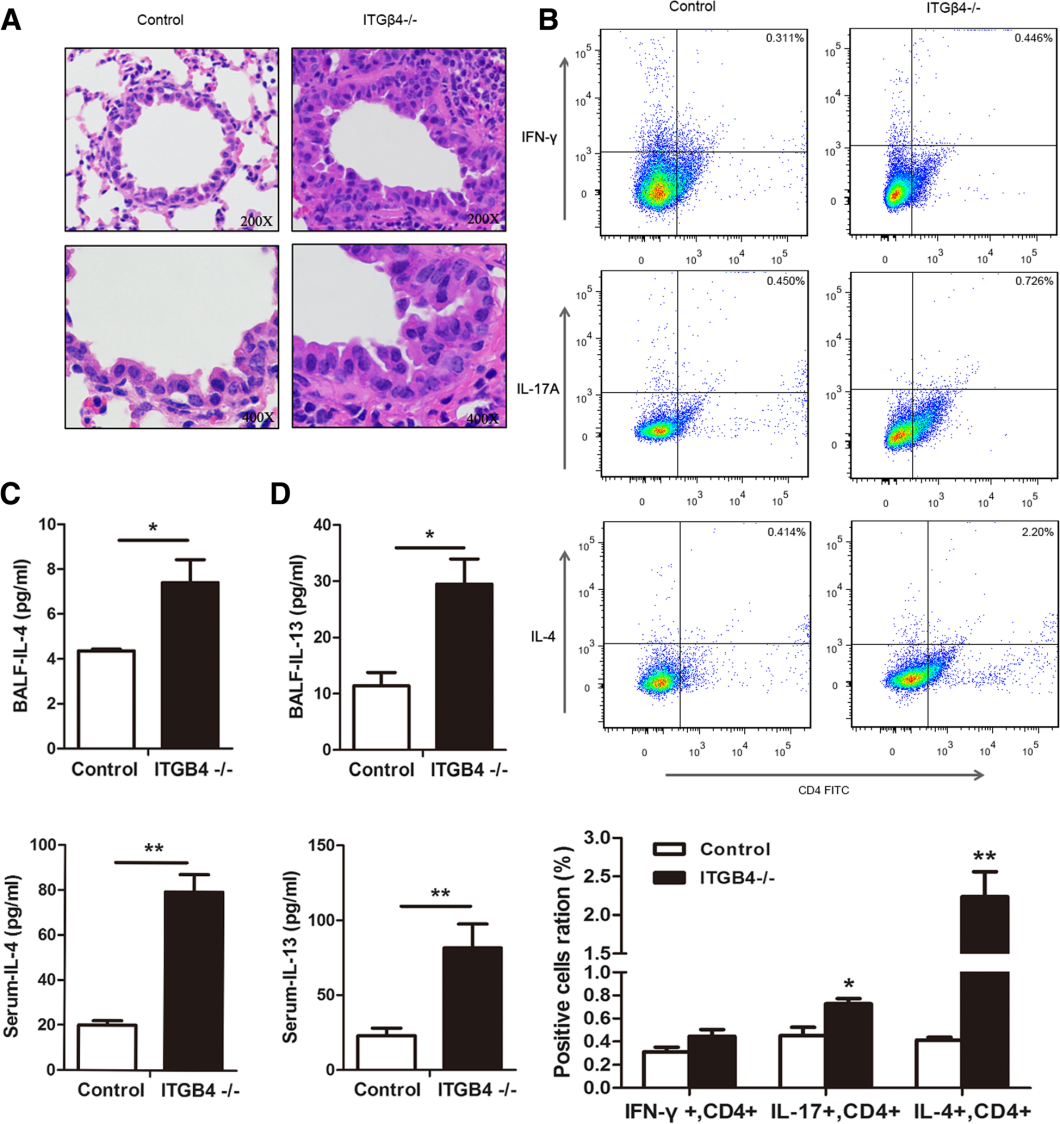
Peripheral inflammation increases in ITGB4^−/−^ mice. **a** H & E staining of mouse lung tissues from the control group and the ITGB4^−/−^ group. All images were obtained at × 200 (upper) and × 400 (lower) magnification. **b** Lung tissues Th1 cells (%), Th2 cells (%) and T17 cells (%) determined by flow cytometry in mice from the control group and the ITGB4^−/−^ group. Th2 cells in ITGB4^−/−^ group showed a significant increase. **c**, **d** Measurements of IL-4 levels (**c**) and IL-13 levels (**d**) in the BLAF and serum by ELISA. The IL-4 and IL-13 in ITGB4^−/−^ group showed significant increase compared to that in the control group. The data are expressed as the mean ± SEM of four mice in each group. *p < 0.05, **p < 0.01

Hematoxylin and eosin (H&E) staining of lung tissues from the ITGB4^−/−^ mice demonstrated increased mucous secretion, increased mucosal folds, visible epithelial fractures, epithelial cell shedding, and mild bronchiole smooth muscle hypertrophy, as well as bronchial wall and basement membrane thickening and irregularities in its shape (Fig. 6a). Excessive inflammatory cells in lung tissue from ITGB4^−/−^ mice were found to infiltrate into the bronchial submucosa, bronchial, and perivascular spaces. (Fig. 6a).

Flow cytometry analysis showed that the number of Th17 cells and Th2 cells were greater in ITGB4^−/−^ mice than control mice, especially Th2 cells (Fig. 6b, *p* < 0.01). However, there was no significant difference in the number of Th1 cells between the two groups.

Consistent with the observation of elevated serum IL-4 and IL-13 in patients experiencing asthma, the ITGB4^−/−^ mice showed significantly enhanced IL-4 levels (Fig. 6c, BALF *p* < 0.05; serum *p* < 0.01) and IL-13 levels (Fig. 6d, BALF *p* < 0.05; serum *p* < 0.01) measured by ELISA.

### Inflammation of the central nervous system increase in ITGB4^−/−^ mice

**Fig. 7:**
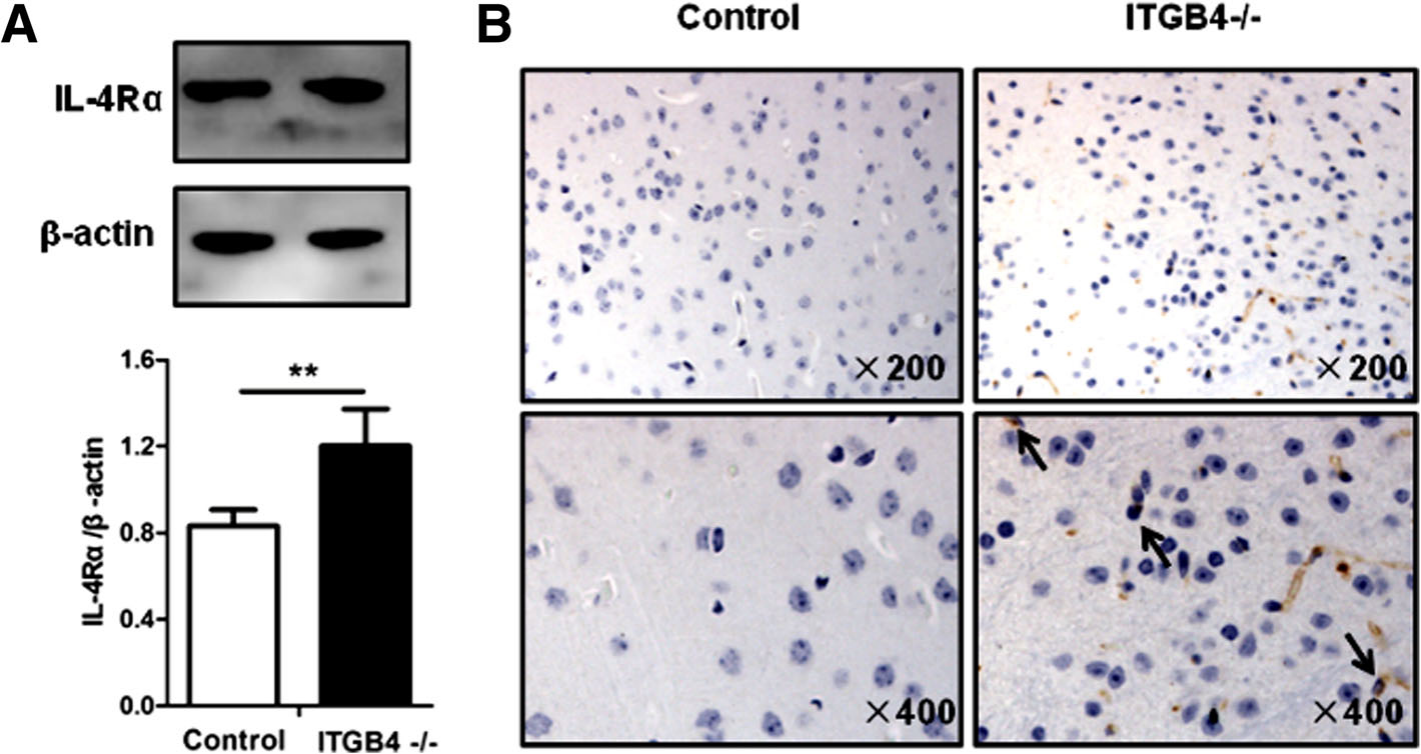
Activated cerebral vascular macrophage increases in ITGB4^−/−^ mice. **a** Representative western blot analysis of IL-4Rα levels of the circumventricular organ and quantification relative to β-actin in control mice and ITGB4^−/−^ mice. **b** Immunoreactivity of IL-4Rα in choroid plexus of mice in the control group and the ITGB4^−/−^ group. All images were obtained at × 200 (upper) and × 400 (lower) magnification. All data are presented as mean ± SEM of four mice in each group. **p < 0.01

To determine the route of peripheral immune information into the central nervous systems, we examined the expression level of IL-4 receptor alpha chain (IL-4Rα) on choroid plexus (CP), an important area of circumventricular organs (CVOs) where the blood–brain barriers (BBBs) are deficient. The IL-4Rα expression level, determined by western blot assays, on the CP of the ITGB4^−/−^ mice, was significantly higher than that in the control mice (Fig. 7a, *p* < 0.01). Consistent with the western blot results, immunohistochemical (IHC) staining of mouse CP also showed more IL-4Rα-positive cells in the ITGB4^−/−^ mice than those in the control mice (Fig. 7b).

**Fig. 8:**
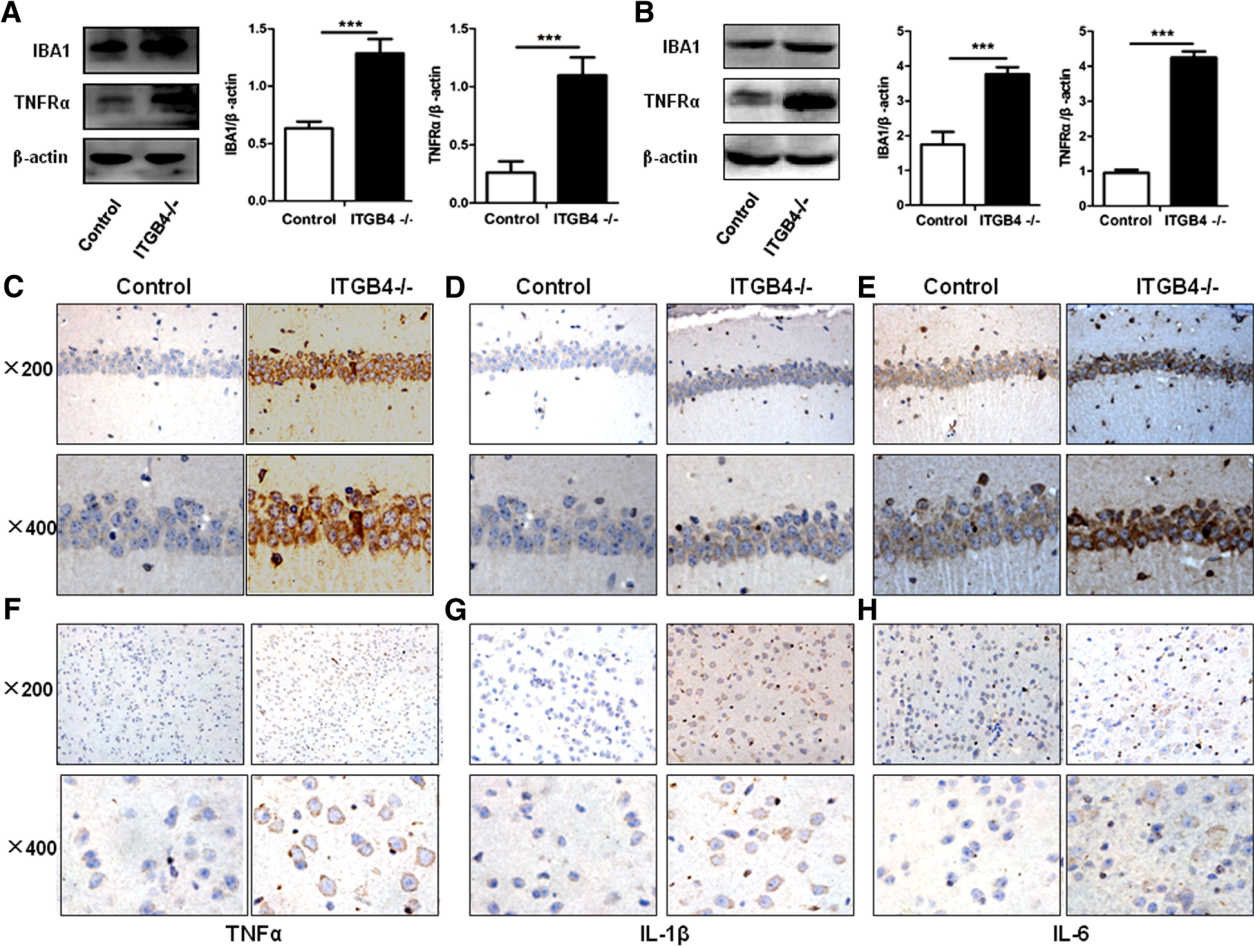
Central nervous inflammation enhanced in ITGB4^−/−^ mice. **a**, **b** IBA1 and TNFRα proteins expression in the hippocampus (**a**) and prefrontal cortex (**b**) determined and quantified by western blot against the expression of β-actin. The ITGB4^−/−^ mice demonstrated significantly higher levels of IBA1 and TNFRα than in the control. **c**, **d**, **e** Immunohistochemical staining was performed to assess the expression of TNFα (**c**), IL-1B (**d**) and IL-6 (**e**) in the hippocampus. All images were obtained at × 200 (upper) and × 400 (lower) magnification. **f**, **g**, **h** Immunohistochemical staining was performed to assess the expression of TNFα (**f**), IL-1β (**g**) and IL-6 (**h**) in the prefrontal cortex. All images were obtained at × 200 (upper) and × 400 (lower) magnification. All data are presented as mean ± SEM of four mice in each group. ***p < 0.001

To further define the microglia as an important interface in transferring information from the peripheral to central nervous system, we used western blot to examine the expression level of IBA1. The ITGB4^−/−^ mice showed significantly enhanced IBA1 expression levels in the hippocampus (Fig. 8a, *p* < 0.001) and prefrontal cortex (Fig. 8b, *p* < 0.001). Moreover, in the ITGB4 deficient group, the expression levels of inflammatory cytokines TNFα (Fig. 8c, f), IL-1B (Fig. 8d, g), and IL-6 (Fig. 8e, h), mainly synthesized and released by microglia in the brain, were higher than those in the control group in the hippocampus (Fig. 8c–e) and the prefrontal cortex (Fig. 8f–h) when tested by immunohistochemical (IHC) staining. In addition, TNFRα was found to increase significantly in ITGB4^−/−^ mice compared with control mice (Fig. 8a, hippocampus and Fig. 8b, prefrontal cortex; *p* < 0.001 respectively).

### Pathological changes in the nervous system increased in ITGB4^−/−^ mice

**Fig. 9:**
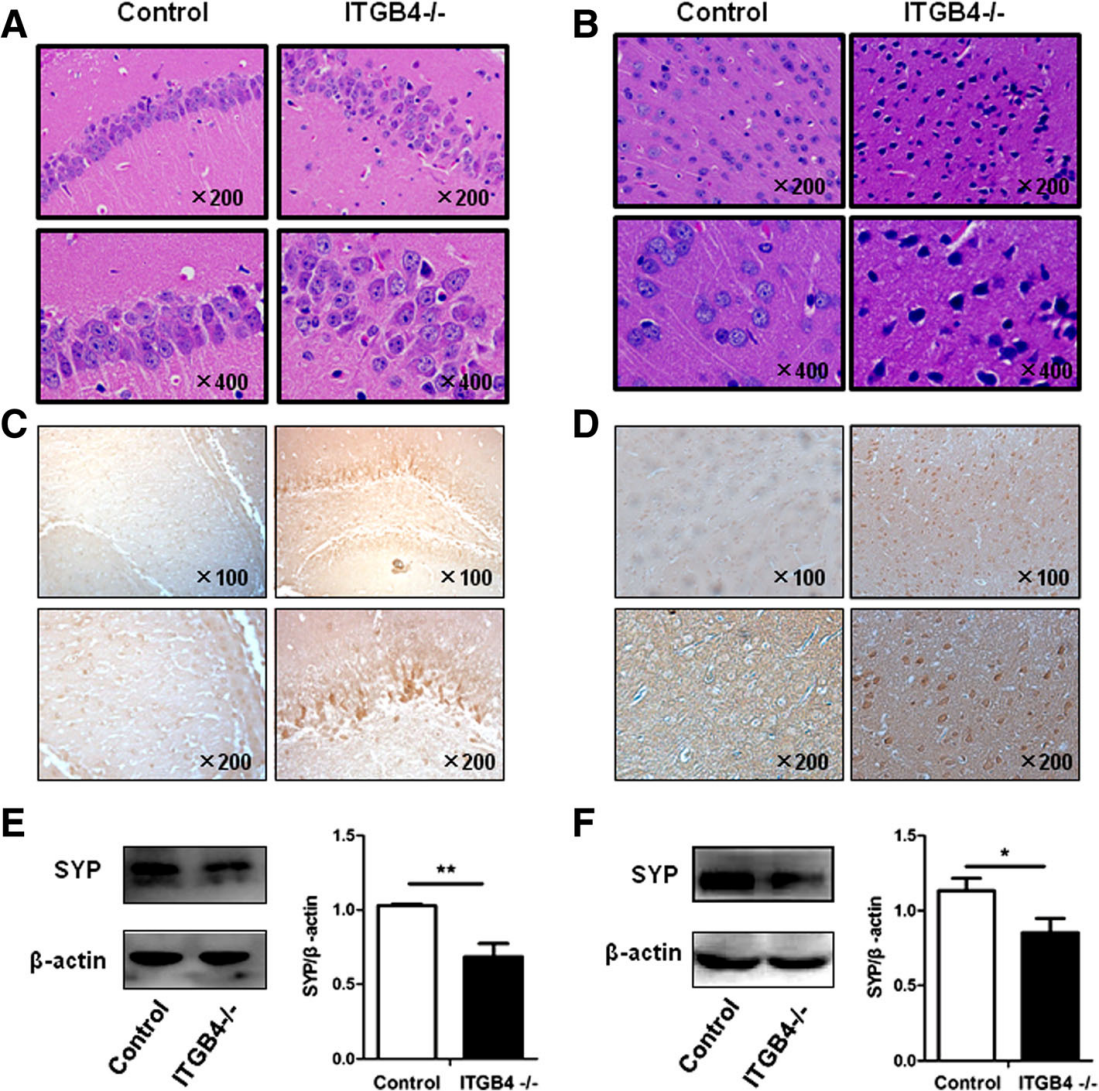
Pathological changes in the hippocampal and prefrontal cortex were found in ITGB4^−/−^ mice. **a**, **b** H&E staining of mouse hippocampus (**a**) and prefrontal cortex (**b**) from the control group and the ITGB4^−/−^ group. All images were obtained at × 200 (upper) and × 400 (lower) magnification. **c**, **d** Apoptosis assay of mouse hippocampus (**c**) and prefrontal cortex (**d**) from the control group and the ITGB4^−/−^ group. All images were obtained at × 100 (upper) and × 200 (lower) magnification. **e**, **f** SYP protein expression in the hippocampus (**e**) and prefrontal cortex (**f**) determined and quantified by western blot normalized against the expression of β-actin. All data are presented as mean ± SEM of four mice in each group. *p < 0.05, **p < 0.01

To investigate the high levels of inflammatory cytokines that could act on neurons to result in pathologic changes, we examined the change of neuronal structure and function, synaptic transmission and plasticity, and apoptotic levels in hippocampus and prefrontal cortex. H&E staining of the hippocampus (Fig. 9a) and the prefrontal cortex (Fig. 9b) from ITGB4^−/−^ mice showed neuron arrangement loosening; nucleus shrinkage, structure fuzzy and deep staining. Apoptosis analysis manifested that elevated numbers of necrotic neurons in the hippocampus (Fig. 9c) and the prefrontal cortex (Fig. 9d) were observed in the ITGB4^−/−^ mice when compared to the control mice. Synaptophysin (SYP), a protein that can be used to reflect synaptic transmission and synaptic plasticity, was found to decrease prominently in ITGB4^−/−^ mice (Fig. 9e, hippocampus, *p* < 0.01 and Fig. 9f, prefrontal cortex, *p* < 0.05).

## Discussion

The present study verified that airway inflammatory is the important incentive for the BD-like behavior during asthma pathogenesis. ITGB4 seems to be a critical participant in the induction of airway inflammation and BD-related behavior. Bronchial epithelial ITGB4 knocked out from the embryonic stage could induce systematically chronic inflammation, microglial activation to secrete neuro-inflammatory cytokines probably through the circumventricular organs and the choroid plexus, and further pathophysiologic changes in the brain to result in BD-like behavior. These findings may help to provide a new animal model for studying the comorbidity of asthma and BD as well as a new avenue for treatment.

Accumulating clinical studies showed that there was significantly high prevalence of BD in asthmatic patients. However, few animal models were used to study the linkages and underlying mechanism between asthma and BD. It is interesting that bronchial epithelial ITGB4 conditional knockout mice presented BD-like behavior obviously. In behavioral test, ITGB4^−/−^ mice showed an increased locomotor activity and decreased habituation in the open-field test, which are typical symptoms of mania and ADHD [39, 41]. Several clinical studies observed that bipolar patients exhibit a greater response to D-AMPH [42], showing increased activity in manic episodes of BD [43], while ADHD patients exert calming response to D-AMPH [44]. As expected, ITGB4^−/−^ mice treated with D-AMPH in the present study showed increased locomotor activity, indicating that the enhanced sensitivity of ITGB4^−/−^ mice to d-amphetamine is related with mania rather than ADHD.

Moreover, the behavioral analysis revealed increased exploration as measured by center time duration in the 5-min open-field test in ITGB4^−/−^ mice. Besides increased exploration, the increased time spent in the center in ITGB4^−/−^ mice, as well as increased entry into and time in the open arms in the elevated plus maze [45] could be related to the mice’s reduced anxiety/increased risking behavior [46, 47], a mania-linked characteristic in manic patients [48, 49]. These findings provide further consistency to increased object interactions in patients with BD [47].

To define the depressive-like behavior of ITGB4^−/−^ mice, we utilized the forced swim test, one of the most commonly used model of behavioral despair. We found an increased immobility time in ITGB4^−/−^ mice, indicating depressive-like behavior in these mice. Anhedonia, a decreased ability to experience pleasure, is also a symptom of depressive-like behavior [50, 51], and reduced sucrose consumption is usually used as an indicator of depression-related anhedonia [51]. Although no significant difference was found in sucrose preference between ITGB4^−/−^ mice and WT littermates, the sucrose consumption decreased after restraint stress in ITGB4^−/−^ mice. Stress plays an acknowledged role in precipitating psychotic episodes in bipolar disorder [52]. Many stressors including psychological, hormonal, and pharmacological that induce modest, transient perturbations in healthy individuals are able to induce mood episodes in individuals with bipolar diathesis [53, 54]. To further affirm this behavioral phenotype, learned helplessness test, an important model to measure helplessness or despair involving sub-chronic stress, was conducted on ITGB4^−/−^ mice and WT littermates. We found that ITGB4^−/−^ mice in the FS group show more escape failures than control animals, suggesting that ITGB4^−/−^ mice are more impaired in learned helplessness. These findings indicate that the ITGB4^−/−^ mice are impressionable to the depression effects of stress.

This study detects hyperlocomotion, psychostimulants sensitivity, anxiolytic-like behavior, and stress-induced depression in ITGB4^−/−^ mice, which were analogous to some features of human BD characterized by an episodic recurrent pathological disturbance in mood ranging from severe depression to extreme mania [55]. In previous reports, evidences reveal that both asthma and BD share common immune dysfunction [1, 56]. And ITGB4 deficiency may play an important role in airway inflammation of asthma patients [19]. Exhilaratedly, our findings revealed that ITGB4^−/−^ mice predominantly exhibited Th2-type inflammation, which was the pivotal characteristic of asthma [57]. IL-4 and IL-13, the major cytokine of Th2-type inflammation, were very highly expressed in bronchoalveolar lavage fluid and blood of ITGB4^−/−^ mice. Meanwhile, IL-4 was also reported elevated immune activation in both manic and depressive state [14, 58]. Combined with previous experimental results that ITGB4^−/−^ mice presented BD-like behavior in our study, these findings verified that Th2 inflammation played a critical role in the association between asthma and BD in ITGB4^−/−^ mice.

As we all know, the effect of IL-4 signaling is mediated through the IL-4 receptor alpha chain (IL-4Rα), which dimerizes either with the common gamma chain (CD132) or with the IL-13 receptor alpha 1 (IL-13Rα1) chain [59]. A number of cell types including macrophages, neurons, astrocytes, and microglia express the IL-4Rα and can respond to IL-4 signaling [59, 60]. In ITGB4^−/−^ mice, IL-4Rα was found highly expressed in choroid plexus (CP), an important area of circumventricular organs (CVOs) with leaky blood–brain barriers (BBBs). Circumventricular organ macrophages responding to IL-4 could release pro-inflammatory cytokine, which then leakage into the brain to promote the production of a second wave of cytokines by microglial cells [61]. Microglia, resident macrophage-like immune cells in the CNS, play a critical role in both physiological and pathological conditions, including restoring the homeostasis of the CNS and driving the neuro-inflammatory response of neurodegenerative disorders, respectively [62]. We really found that ITGB4^−/−^ mice showed a significant increase of microglial and pro-inflammatory cytokines such as IL-1β, IL-6, and TNF-α.

Furthermore, some researches have implicated immune factors in brain development and plasticity [63]. Microglia promote pro-inflammatory responses with excess tumor necrosis factor (TNF-α), interleukin-1β (IL-1β), inducible nitric oxide synthase (iNOS), and reactive oxygen species (ROS) production [64], contributing to neural network dysfunction. Thus, it is possible that inflammation and immune activation could affect brain regions involved in the progress and variation in symptom levels in bipolar disorder. IL-1β is widely distributed in the brain, particularly in the hippocampus and hypothalamus [65]. Consistent with previous results, IL-1β was highly expressed in the hippocampus in ITGB4^−/−^ mice. Subsequent animal studies have indicated that high levels of IL-1β can act on hippocampal neurons to inhibit synaptic strengthening and LTP [66, 67], which was conformed by the decrease of SYP in ITGB4^−/−^ mice. A postmortem study on the prefrontal cortex revealed that the IL-1β protein and mRNA levels were significantly higher in the patients with BD [68]. This result is in line with our findings that IL-1β expression increased obviously in the prefrontal cortex of ITGB4^−/−^ mice. In the last decade, the importance of cytokines in neuronal survival [69] was recognized in the pathophysiology of BD. ITGB4^−/−^ mice did show neuron damage and increased neuron apoptosis. In addition, many studies have also reported increased TNF-α and IL-6 levels in acute phases of mania and depression compared to the controls [14, 58, 70]. Pro-inflammatory cytokine TNF-α and IL-6 in neurotransmitters, neuroplasticity, and neuronal survival [71–73] was linked to the pathophysiology of BD. Interestingly, our experiments showed that the expression levels of TNF-α and IL-6 were higher in hippocampus and prefrontal cortex of the ITGB4^−/−^ mice than the control mice. These results support the concept that BD have been associated with changes of the immune response.

## Conclusions

Our results suggested that ITGB4 knockout induced chronic inflammation mediated the comorbidity of asthma and BD. Notably, the immune-to-brain communication was realized by the production and action of inflammation cytokines that propagate from the circumventricular organs and the choroid plexus into the brain. Microglial cells in the brain were activated by the incoming information to produce pro-inflammatory cytokines which contribute to neural network dysfunction and then trigger BD occurrence. It provided additional evidence of the potential molecular mechanisms of the asthma co-existence with BD and explained why patients with asthma frequently suffer from BD.

## Abbreviations

ADHD: Attention-deficit hyperactivity disorder
AIH: Amphetamine-induced hyperactivity
BALF: Bronchoalveolar lavage fluid
BD: Bipolar disorder
D-AMPH: d-Amphetamine
ELISA: Enzyme-linked immunosorbent assay
EPM: Elevated plus-maze test
FST: Forced swimming test
IBA1: Ionized calcium binding adapter molecule 1
IL-13: Interleukin-13
IL-1β: Interleukin-1β
IL-4: Interleukin-4
IL-6: Interleukin-6
ip: Intraperitoneal
ITGB4: Integrin β4
ITGB4^−/−^: ITGB4 deficient
LPT: Learned helplessness test
OFT: Open-field test
SPT: Sucrose preference test
SYP: Synaptophysin
TNF-α: Tumor necrosis factor-α
